# Efficacy of a Novel Rigenase^®^ and Polyhexanide (Fitostimoline^®^ Septagel) Hydrogel Device for the Treatment of Vulvovaginitis Symptoms: Cross-Sectional Analysis of a National Survey and Prospective Observational Study

**DOI:** 10.3390/medicina59112004

**Published:** 2023-11-15

**Authors:** Gaetano Riemma, Giampaolo Mainini, David Lukanović, Gaetano Scalzone, Lucia Sandullo, Maria Teresa Schettino, Maria Giovanna Vastarella, Mattia Dominoni, Gorizio Pieretti, Pasquale De Franciscis, Mario Passaro, Marco Torella

**Affiliations:** 1 Department of Woman, Child and General and Specialized Surgery, University of Campania “Luigi Vanvitelli”, 80138 Naples, Italy; gaetano.riemma@unicampania.it (G.R.); drgscalzone@gmail.com (G.S.); luciasandullo@virgilio.it (L.S.); mariateresa.sche@gmail.com (M.T.S.); mariagiovannavastarella@hotmail.it (M.G.V.); gorizio.pieretti@unicampania.it (G.P.); pasquale.defranciscis@unicampania.it (P.D.F.); 2 ASL Napoli 3 Sud, 80056 Naples, Italy; 3 Department of Gynecology, Division of Gynecology and Obstetrics, Ljubljana Medical Center, 1000 Ljubljana, Slovenia; david.lukanovic@mf.uni-lj.si; 4 Obstetrics and Gynecology Unit, Fondazione IRCCS Policlinico San Matteo, 27100 Pavia, Italy; matti.domino@gmail.com; 5 ASL Caserta, 81100 Caserta, Italy; pm70doc@gmail.com

**Keywords:** vulvovaginitis, bacterial vaginosis, local therapy, non-antibiotic treatments, vaginal burning

## Abstract

*Background and Objectives*: Signs and symptoms of vulvovaginitis, especially when recurrent, have a significant impact on a woman’s quality of life. The aim of this study was to survey gynecologists about their habits regarding the treatments of the pathology and to evaluate the efficacy of a novel vaginal hydrogel composed of wheat extracts and polyhexanide aimed at reducing vulvovaginitis symptomatology. *Materials and Methods*: A cross-sectional analysis of a national survey using 155 Italian gynecologists and a prospective, open-label, observational study were carried out in 75 outpatient clinics across Italy. Pre- and postmenopausal women with suspicion of vulvovaginitis due to at least four of the following symptoms (leucoxanthorrhea, bad odor from genitalia, vulvovaginal dryness, petechiae, burning, and pruritus) while waiting for microbiological swab analysis were included and treated with one hydrogel application every 3 days for 1 week. Primary endpoint was the complete resolution of symptomatology. *Results*: The pre-study survey reported that, for most clinicians, local or oral treatment (65.7% and 82.8%, respectively) with antibiotics or antifungals is used very often. Therefore, we proceeded to carry out an observational study. Overall, 615 (362 of fertile age and 253 in postmenopause) women were included in this study. At the 28th follow-up examination, complete resolution of symptomatology was achieved in 578/615 (94.1%; *p* < 0.001) within 12.72 ± 6.55 and 13.22 ± 6.33 days for those of fertile age and in postmenopause, respectively (*p* = 0.342). All of the evaluated symptoms were significantly reduced after treatment (*p* = 0.001) without differences according to the patient’s menopausal status. A slightly significant reduction in *Gardnerella Vaginalis* (*p* = 0.040) and *Candida Albicans* (*p* = 0.049) was found after treatment. No patient reported side effects, adverse reactions, or discontinued therapy. *Conclusions*: This pilot study showed that a hydrogel based on Rigenase^®^ (wheat extract) and polyhexanide could be a promising treatment for the relief of vulvovaginitis symptoms. However, these results are limited by the absence of a control group. Additional comparative and randomized controlled trials between the hydrogel and other non-antibiotic devices as well as local antibiotic therapy should be performed to increase the validity of the findings.

## 1. Introduction

Vulvovaginitis is an inflammatory condition involving the vulva and the vagina [1]. While it is generally caused by infection, this, especially after menopause, may be the consequence of a hormonal imbalance [2]. Typical symptoms are burning, pruritus, dyspareunia, pain, leucoxanthorrhea, and vaginal discharge [3].

There are different types of vaginitis, which have other causes, symptoms, and treatments. The most common types are bacterial vaginosis, which affects approximately 22 to 50% of symptomatic women, Candida-species vulvovaginitis (17% to 39%), and Trichomonas vaginalis (4% to 35%), and, less frequently, other specific pathogens cause it, including Mycoplasma and Ureaplasma [4,5].

While each type of vulvovaginitis has its own specific treatment, according to national and international guidelines, diagnosis may sometimes be time-consuming, and alternative treatments that could alleviate symptoms while preventing the worsening of the causative factor may be useful [6,7]. To understand whether this leads to an overuse of local or systemic treatment with antibiotics/antifungals, our clinical study was preceded by a survey performed by Italian gynecologists. The survey confirmed that there is an unmet need to treat these cases of vaginitis where an immediate clinical diagnosis is unfeasible.

Fitostimoline^®^ Septagel (Damor Pharmaceuticals, Naples, Italy) is a novel medical device containing Rigenase^®^, a proprietary wheat extract from Triticum Vulgare, plus polyhexanide, an antiseptic active on both bacteria and yeasts, in a novel vaginal hydrogel formulation with anti-inflammatory, antioxidant, and wound-healing properties [8]. Due to the adhesive properties of the novel hydrogel formulation, the device has a posology of one application every three days. For all of these reasons, this medical device seems an excellent candidate to be used as an alternative or adjuvant therapy to antimicrobic agents for relieving the symptoms of vulvovaginitis [9].

We, therefore, decided to perform an observational, open-label, prospective study evaluating the effects of treatment with Fitostimoline^®^ Septagel in a large cohort of pre- and postmenopausal women with vulvovaginitis.

## 2. Materials and Methods

This study was designed following the Helsinki Declaration and conformed to the Committee on Publication Ethics (COPE) guidelines. The protocol design, collection, analysis, and interpretation of data, drafting, and subsequent revisions followed the Strengthening the Reporting of Observational Studies in Epidemiology (STROBE) Statement guidelines for reporting observational studies [10]. This study was approved by the reference ethics committees of one of the study sites (University of Campania Ethical Committee, protocol code no. 728-26 November 2019).

### 2.1. Pre-Study Survey

The clinical study was preceded by a survey performed using gynecologists in Italy to understand which was the correct approach for patients with vaginitis when the clinical examination did not allow for the diagnosis of the pathogen responsible for the symptoms.

Gynecologists who reported that they routinely treated vulvovaginitis as part of their gynecologic practice participated in this Internet-based cross-sectional study.

Two supervisors (GM and MP) created the survey, and a central panel of foreign researchers evaluated its reliability and content validity.

The survey was delivered via email and administered using SurveyMonkey’s web-based program (SurveyMonkey, 2020). The survey completion constituted an agreement to participate; participation was voluntary and no individual participant could be identified.

To maintain participant confidentiality, only the location of the gynecologist was reported for demographic reasons. The answers of the gynecologists were depicted as an accurate report of their most recent clinical routine.

Before concluding that the invited gynecologists were unwilling to participate, a 14-day response period was given. Clinicians received the survey via email up to three times before being excluded from the poll. Using Internet data, responses could not be linked to specific people.

If the results had supported the idea that local treatment with antibiotics or antifungals is used very often in these patients, contrary to what is suggested by clinical guidelines, we would have proceeded to the next observational part of this study.

### 2.2. Observational Study

This prospective, observational, open-label, multicentric study was conducted in 75 gynecological outpatient services distributed in Italy, supervised by two main centers (ASL Napoli 3 Sud. Naples, Italy, and AOU Luigi Vanvitelli, Naples, Italy).

From June 2022 to October 2022, all patients presenting with symptoms of vulvovaginitis to whom treatment with Fitostimoline^®^ Septagel had been prescribed were enrolled in this study.

Before enrollment, written informed consent was obtained from all of the participants. In the informed consent form, each participant in this study received information about the methods and provided their agreement to enable data to be anonymized, collected, and analyzed for research.

Patients were included according to the following criteria: female subjects, good knowledge of Italian language, age > 18 years, non-pregnant, non-breastfeeding, presence of at least four among the following six symptoms: leucorrhea, defined as thick, white discharge from the internal genitalia, xanthorrhea, defined as thick yellowish discharge, and leucoxanthorrhea, defined as white/yellowish genitalia discharge, “bad smell” coming from the genitalia (bad odor), and subsequently vulvovaginal dryness, vulvar and vestibular pain, vulvovaginal petechiae, burning, and itching (pruritus).

After a vaginal microbial swab, the signs and symptoms were firstly evaluated through a clinical interview conducted by an assistant and registered using computer-based, dedicated data management software. Subsequently, clinically retrievable signs were re-evaluated for confirmation via a careful gynecological examination by a different unbiased clinician.

The presence of leucorrhea, xanthorrhea, or leucoxanthorrhea was reported by the patient and confirmed via speculum-based vaginal exploration. For bad genital odor and vulvovestibular pain, signs were confirmed using the whiff test (placing the vaginal discharge on a slide with 10% potassium hydroxide solution) and the vulvodolorimeter approach (pressure pain thresholds in the vulvar area evaluated in 23 defined locations) [11], respectively. Vulvovaginal dryness, burning, and itching were subjectively evaluated by the patient.

All of the subjective and objective items were standardized using a semiquantitative scale from 0 to 3, in which signs and symptoms were categorized as follows: 0 = absence; 1 = mild; 2 = moderate; and 3 = severe.

Exclusion criteria were current or recent (less than three months) antibiotic/antifungal therapy (local or systemic) or treatment with prebiotics and/or probiotics and/or anti-inflammatories; neoplastic diseases of the genital tract; any systemic disease potentially interfering with the vulvovaginal mucosa; allergy; intolerance; or known reaction related to the compound’s ingredients and excipients.

Each patient applied the hydrogel inside their vagina at bedtime once every three days for one week (3 applications). The patients were carefully advised on how to precisely apply the hydrogel. With regard to their first usage, they had to perforate the gel tube, using the perforator contained in the top of the end cap, and subsequently screw in the single-use vaginal applicator. Afterwards, the gel tube needed to be squeezed until the applicator had been filled. Subsequently, the women had to unscrew the applicator from the tube, introduce it into the vagina, and depress the plunger until it reached the end of its stroke. The hydrogel packaging contained the minimum amount of single-use applicator for the study period.

To avoid discrepancies and biases due to factors not related to the therapy regimen, patients were asked to avoid sexual intercourse for the study period.

Patients were re-evaluated 28 days after the first visit using the same methodology of the first examination. Symptoms were registered and clinically assessed again, and a second vaginal swab was carried out.

The patients whose vulvovaginitis symptoms and signs were resolved (total symptom score equal to or less than 2) after therapy were defined as having “complete resolution” and this was considered to be the primary effectiveness outcome. Subsequently, every sign/symptom was evaluated separately and according to the pre- or postmenopausal status of the woman. These were considered to be the secondary outcomes of this study.

Patients were also asked to indicate the day of their symptomatology’s disappearance and any possible side effects (systemic or gynecological) that could be related to the use of the hydrogel.

### 2.3. Statistical Analysis

Stata 14.1 (Stata Corp., College Station, TX, USA) and GraphPad Prism 9.5 (GraphPad, La Jolla, CA, USA) were used to conduct the statistical analysis.

A one-sided test analysis determined that a sample size of 214 patients would have a power of 90% in rejecting the hypotheses of nonequivalence or inferiority, respectively.

For the main study, descriptive data were assembled. Means and standard deviations (SDs) for symmetrically distributed continuous variables were given, and the *t*-test was used to examine the mean differences. The representation of dichotomous data was in absolute numbers and percentages. Where applicable, Fisher’s exact or the chi-square test for multiple comparisons was used to assess differences in the proportions between the groups. The threshold for statistical significance was set at a *p*-value (*p*) less than 0.05.

## 3. Results

### 3.1. Pre-Study Survey

**Figure 1:**
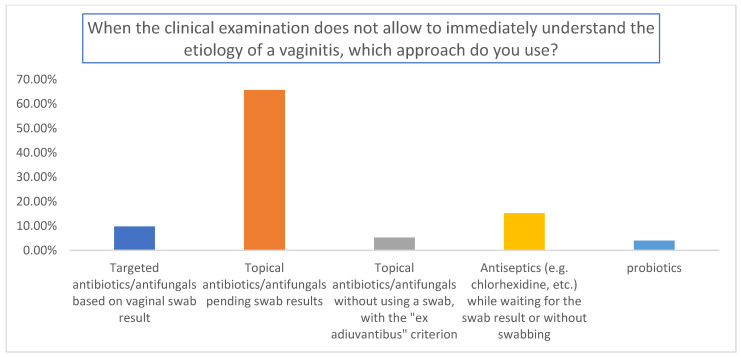
Evaluation of the approach used by the gynecologist when the clinical examination did not allow them to immediately understand the etiology of the vaginitis.

**Figure 2:**
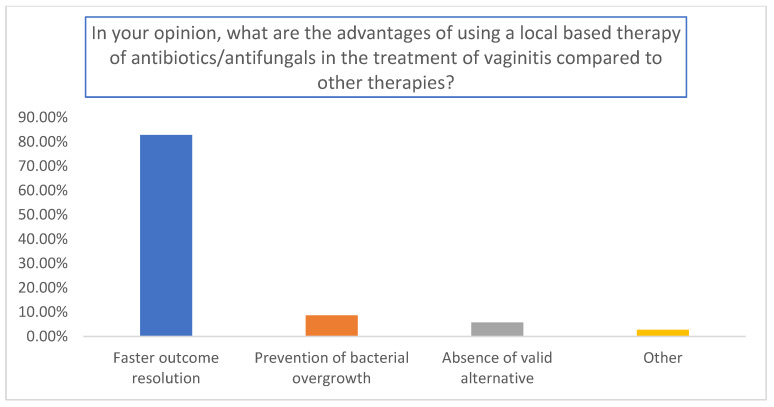
Evaluation of the advantages of using antibiotics/antifungals in comparison to other therapies.

**Figure 3:**
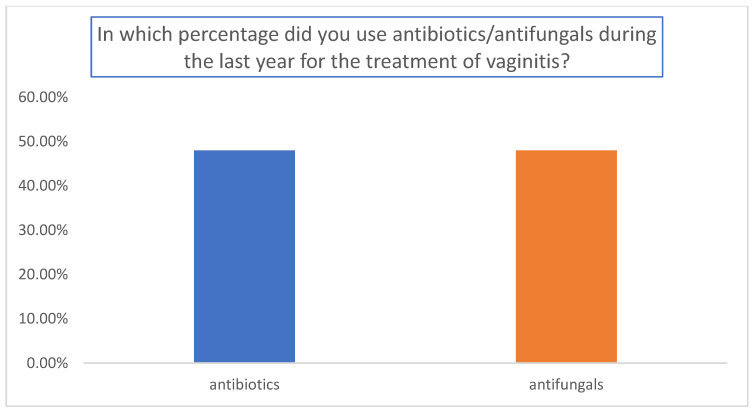
Percentages of antibiotics and antifungals used during the last year for the treatment of vaginitis.

The results are summarized in Figure 1, Figure 2 and Figure 3. A total of 155 gynecologists responded to the questionnaire. Among them, 65.7% used topical antibiotics/antifungals pending swab results (Figure 1); 82.8% used antibiotics for a faster resolution of outcomes (*p* < 0.01 relative to the other answers) (Figure 2), and the percentages of antibiotics and antifungals were comparable in the analyzed cohort (Figure 3).

### 3.2. Observational Study

After applying the inclusion and exclusion criteria, 615 women were included in the analysis: 362 were of a fertile (58.86%) age and 253 were of a postmenopausal (41.14%) age, with an average time-to-menopause of 9.52 ± 8.18 years (Table 1).

Leucoxanthorrhea and bad odor were reported more often (by 94.31% and 91.87% of the patients, respectively). Pruritus and dyspareunia were also very frequently reported (84.23% and 67.97%, respectively), while the presence of dryness, petechiae, and burning was slightly less frequent (55.28%, 42.28%, and 58.54%, respectively). After treatment, most women reported a statistically significant complete resolution of their symptomatology (578/615; 94.1%), while 5.9% (37/615; *p* < 0.001) had a minor improvement. In most cases, an improvement in symptoms was first observed during the first 14 days of this study (12.72 ± 6.55 vs. 13.22 ± 6.33 days for those of fertile age and in postmenopause, respectively; *p* = 0.342).

Detailed differences between symptoms before and after 1-week of treatment, as evaluated at the 28th-day gynecological examination, are reported in Table 2. There was a significant decrease in each reported score.

Similarly, all of the symptoms were significantly reduced in both premenopausal and postmenopausal women, with no substantial differences between the two cohorts (Table 3).

The results of the microbiological swab analysis before and after the hydrogel application showed a reduction in overall positivity, with a slightly significant reduction for *Gardnerella Vaginalis* (*p* = 0.040) and *Candida Albicans* (*p* = 0.049) after treatment (Table 4).

Regarding side effects and complications, no woman had to discontinue therapy due to adverse reactions, complexity of the hydrogel application procedure, or dissatisfaction.

## 4. Discussion

Our cross-sectional analysis of a national survey confirmed the need for a better non-antibiotic/non-antifungal treatment for vaginitis for the symptomatic relief of cases where a clear clinical diagnosis is unfeasible, to be used while waiting for the microbiological analysis. Most clinicians still use antibiotic empirical therapy while waiting for swab test results, which is not recommended by international guidelines. However, using antibiotics to treat the condition is linked to significant failure and recurrence rates. These may be linked to antibiotic resistance, the difficulty to remove polymicrobial biofilms, the failure to restore an acidic pH and the lactobacillus-dominated commensal flora, and the failure to eliminate the polymicrobial biofilms. Therefore, in order to prevent and treat vulvovaginitis more effectively, it is imperative to research alternatives to or in addition to current medications. Antimicrobial agents (including antiseptics and natural chemicals) might be used as alternatives [7]. Considering the rising of antibiotic resistance and the efforts in avoiding the excessive use of antibiotics, the use of non-antibiotic devices that show a significant improvement in symptoms is a paradigm-shift approach. The more literature that is provided on the efficacy of non-antibiotic compounds in symptom relief while waiting for a swab result, the most efficacy we can obtain against antimicrobial resistance, and, as a matter of fact, less clinicians will still prescribe empirical antibiotics, leading to a targeted, precise, and less invasive therapy for the woman.

Findings from previous qualitative research are similar to the symptoms of vulvovaginitis, such as repulsive odor and atypical vaginal discharge, that have such a significant influence on women’s lives [12,13]. Similarly, recurring bacterial vaginosis has an emotional trauma-causing effect on women’s lives, in addition to the bacterial symptoms. Due to the physical, mental, and social strain of dealing with recurring vaginosis, women’s employment and personal relationships were simultaneously impacted by an increase in paranoia and a decrease in self-esteem [14].

In this regard, our observational study is a pilot proof-of-concept study suggesting that treatment with Fitostimoline^®^ Septagel could promptly and efficiently achieve relief from symptoms of vulvovaginitis since, after 14 days, almost all patients showed a complete resolution of symptoms, without any side effects, independent from their menopausal status.

Rigenase^®^ is a particular Triticum Vulgare extract that exhibits intense antioxidant activity by maintaining scavenger action against free radicals. By boosting chemotaxis, fibroblastic proliferation, and maturation, it also optimizes the process of tissue regeneration [15]. These characteristics are brought about by increased protein synthesis, proline absorption, and the overexpression of several essential components such as matrix metalloproteinase (MMP)-2, MMP-9, collagen I, and elastin [15,16,17]. Pressure sores, venous leg ulcers, wounds, burns, delayed scarring, dystrophic disorders, and, more broadly, issues with re-epithelialization or tissue regeneration are all treated using this medication [16,18,19]. The medical device Fitostimoline^®^ Septagel was made using Rigenase^®^ and polyhexanide, inhibiting colonization and wound contamination. This polysaccharide from plants can stimulate the manufacture and release of specific proteins from keratinocytes. The bulk of these secreted proteins function as crosstalk effectors and aid tissue regeneration and repair [15]. Rigenase^®^ specifically encourages cell migration and boosts the production of a new extracellular matrix [15].

With the human host, the vaginal microbiota establishes a homeostatic and mutualistic interaction that has a significant impact on both vaginal health and illness. The breakdown of a balanced ecosystem, often referred to as dysbiosis, is caused by differences in internal and/or external variables [20]. Fitostimoline^®^ Septagel’s active ingredient, the cationic polymer polyhexanide, disrupts the stability of the binding of anionic phospholipids to bacterial cell membranes, promoting the formation of a helpful vaginal microbiome, which is intended to reduce the incidence of bacterial or yeast infections [21]. The risk–benefit ratio is higher than with other antibacterial drugs due to its relatively limited interaction with human cells [21,22]. For this purpose, in our study, there were no reports of minor or major adverse reactions or side effects related to the application of the product.

Triticum vulgare extracts are currently used, thanks to their wound-healing properties, in several dystrophic conditions, including diabetic foot ulcers [23], chronic wounds [16], and cutaneous burns [18]. Similarly, previous studies have reported the efficacy of Triticum vulgare extracts in relieving symptoms of gynecological complaints, such as cervical dystrophy, vaginal atrophy related to genitourinary syndrome of menopause, and vulvovaginitis [24,25]. However, the Fitostimoline^®^-based devices used in such studies were designed to be used in unspecific body regions. Nonetheless, the previous version of the product had already been reported as being equally effective compared to benzydamine hydrochloride in both vaginal ovules and a combined (vaginal cream plus vaginal washing) approach in improving complaints from vulvovaginal atrophy [25] and vulvovaginitis [24]. Compared to the previously available formulation, the new vaginal hydrogel comes with the addition of polyhexanide and the new patented single-use vaginal applicator, making Fitostimoline^®^ Septagel specifically designed for the vulvovaginal environment. With the increased user-friendliness, there was not only a significant improvement in the symptomatology, but also no discontinuation of the therapy, since no women stopped the treatment regimen due to dissatisfaction.

The sequential delivery of medications that target many therapeutic goals may be the secret to therapeutic effectiveness for vulvovaginitis [7]. These combined therapeutic regimens include a first phase of treatment consisting of the administration of agents that could eliminate and stop the development of bacterial biofilms, and a second phase intended to restore the physiologic vaginal environment, consisting of the administration of acidifying agents to decrease pH values to below 4.5, or the administration of probiotics [7,26]. This hydrogel could be considered a valid alternative in both phases due to its antimicrobial activity and tissue restoration relative to the combination of Triticum Vulgare extracts and polyhexanide.

### Limitations

Several limitations of this first observational research on the topic should be acknowledged. Firstly, our research is mainly limited by the absence of a control group and the open-label observational design which did not allow us to draw definitive conclusions. The high number of centers involved in the research and the request of a valid treatment for women demanding help for unpleasant gynecological symptoms that were having a clear impact on their quality of life did not allow us to include a control group of women without treatment. For a gynecologist dealing with symptomatic women, it is often a challenging debate as to whether or not a placebo should be provided for a woman that has the urge of solving symptoms that are deeply affecting their quality of life; some experts even describe such an issue as unethical, since, in such a scenario, an improvement in women’s symptoms is the turning point of the research [22]. Still, our observations may represent a good starting point for future randomized controlled trials that should include a placebo group. Moreover, we aim in the very near future to add further comparative studies (e.g., Fitostimoline^®^ Septagel versus other non-antibacterial vaginal devices) to increase the validity of these first insights regarding the alleviation of vaginal discharge and other vulvovaginitis-related complaints. In addition, the reduced incidence of vulvovaginitis pathogens after treatment cannot be directly related to the treatment itself, since their presence in a vaginal swab analysis may also spontaneously decrease or be related to the quantity of the retrieved specimen. Nonetheless, the findings of our study remain highly relevant due to the urgent need to find non-antibiotic, efficient, and cost-effective devices to reduce symptoms related to vulvovaginal inflammation.

However, the large sample size, the inclusion, and the subsequent comparison of women of a fertile age and postmenopausal women represent valid points of strength of our research, adding robustness to the findings and justifying the possibility of additional trials to strengthen evidence on the topic.

## 5. Conclusions

Therefore, we consider the association of Rigenase^®^ and polyhexanide in vaginal hydrogel formulation (Fitostimoline^®^ Septagel) a promising therapeutic innovation for reducing vulvovaginitis signs and symptoms in women of childbearing age and women who have been through the menopause. In uncomplicated cases, its composition might allow for avoiding antibiotic/antimycotic agents, reducing the risk of creating resistance and allowing a prompt recovery from symptoms. However, to increase the certainty of the available evidence, further comparative and randomized trials should be carried out to compare the hydrogel to other antibiotic and non-antibiotic treatments currently used in daily practice.

## Figures and Tables

**Table 1:** Baseline characteristics of women included in the observational study.

	Whole Population (SD)	Fertile Age (SD)	Postmenopausal (SD)	*p*-Value
Age, years	45.32 (14.99)	35.12 (9.01)	59.91 (8.27)	0.001
BMI, kg/m^2^	23.75 (2.88)	21.77 (3.21)	24.01 (2.26)	0.314
Years after menopause	/	/	9.52 (8.18)	
No. white ethnicity	599	355	244	0.723
No. patients	615	362	253	0.254

No.: number of patients.

**Table 2:** Signs and symptoms of vulvovaginitis before and after treatment.

	Before		After		*p*-Value
	Mean	SD	Mean	SD	
Leucorrhea	2.89	0.32	1.00	0.01	0.001
Xanthorrhea	2.84	0.37	1.00	0.06	0.001
Leucoxanthorrhea	2.80	0.40	1.01	0.09	0.001
Bad odor	2.72	0.46	1.01	0.11	0.001
Vaginal dryness	2.69	0.48	1.02	0.14	0.001
Vaginal petechiae	2.72	0.46	1.02	0.13	0.001
Vulvar and vestibular pain	2.77	0.44	1.02	0.14	0.001
Vulvovaginal burning	2.80	0.41	1.02	0.14	0.001
Vulvovaginal pruritus	2.82	0.39	1.01	0.10	0.001

Data reported as mean value and SD (standard deviation) using a standardized semiquantitative scale from 0 to 3.

**Table 3:** Signs and symptoms of vulvovaginitis before and after treatment according to menopausal status.

	Fertile Age				Postmenopause			
	Before		After		Before		After	
	Mean	SD	Mean	SD	Mean	SD	Mean	SD
Leucorrhea	2.88	0.33	1.00	0.07 *	2.91	0.29	1.00	0.01 °
Xanthorrhea	2.82	0.38	1.01	0.07 *	2.87	0.34	1.00	0.00 °
Leucoxanthorrhea	2.78	0.41	1.01	0.13 *	2.81	0.39	1.01	0.11 °
Bad odor	2.70	0.47	1.02	0.16 *	2.74	0.44	1.00	0.06 °
Vaginal dryness	2.66	0.50	1.03	0.13 *	2.73	0.46	1.01	0.11 °
Vaginal petechiae	2.70	0.47	1.02	0.13 *	2.74	0.45	1.02	0.14 °
Vulvar and vestibular pain	2.75	0.46	1.02	0.14 *	2.80	0.41	1.02	0.15 °
Vulvovaginal burning	2.78	0.42	1.02	0.12 *	2.83	0.38	1.02	0.14 °
Vulvovaginal pruritus	2.80	0.40	1.01	0.29 *	2.85	0.37	1.00	0.06 °

Data reported as mean value and SD (standard deviation) using a standardized semiquantitative scale from 0 to 3. * *p* < 0.01 before vs. after treatment when of a fertile age; ° *p* < 0.01 before vs. after treatment in postmenopausal women.

**Table 4:** Microbiological analysis of pathogens from vaginal swabs taken before and after treatment.

	Before		After		*p*-Value
	No.	%	No.	%	
*Gardnerella Vaginalis*	352	57.2	315	51.2	0.040
*Candida Albicans*	173	28.1	142	17.9	0.049
*Trichomonas Vaginalis*	90	14.7	69	11.2	0.089
Total	615	100	526	85.5	0.001

No.: number of patients with positive swab.

## Data Availability

The data presented in this study are available upon request from the corresponding author.
